# Perinatal Outcomes in a Population of Diabetic and Obese Pregnant Women—The Results of the Polish National Survey

**DOI:** 10.3390/ijerph18020560

**Published:** 2021-01-11

**Authors:** Cezary Wojtyla, Pawel Stanirowski, Pawel Gutaj, Michal Ciebiera, Andrzej Wojtyla

**Affiliations:** 1 International Prevention Research Institute—Collaborating Centre, Calisia University, 62-800 Kalisz, Poland; 2 Club 35, Polish Society of Gynecologists and Obstetricians, 02-677 Warsaw, Poland; stanirowski@gmail.com (P.S.); pgutaj@o2.pl (P.G.); michal.ciebiera@gmail.com (M.C.); 3 Department of Oncological Gynecology and Obstetrics, Centre of Postgraduate Medical Education, 00-416 Warsaw, Poland; 4 First Department of Obstetrics and Gynecology, Medical University of Warsaw, 02-015 Warsaw, Poland; 5 Department of Reproduction, Poznan University of Medical Sciences, St, 60-535 Poznan, Poland; 6 Second Department of Obstetrics and Gynecology, Centre of Postgraduate Medical Education, 01-809 Warsaw, Poland; 7 World Institute for Family Health, Calisia University, 62-800 Kalisz, Poland; a.wojtyla55@gmail.com

**Keywords:** gestational diabetes mellitus, diabetes, obesity, pregnancy, cesarean section

## Abstract

Obesity and diabetes increase the risk of complications during gestation and at delivery. The aim of this study was to compare the perinatal outcomes in the populations of diabetic and obese Polish women, based on the results of a national survey performed in years 2012 and 2017, as well as to determine the risk factors of the gestational diabetes mellitus (GDM). Questionnaires from 6276 women were collected. Obese women constituted 5.5% and 7.5% of study population in years 2012 and 2017, respectively. Among women whose pregnancies were complicated by diabetes mellitus, GDM constituted the most common type of glucose intolerance during both time periods (2012: 89% vs. 2017: 85.6%). In the group of obese women an insignificant increase in the rate of induced deliveries was noted (2012: 9.9% vs. 2017: 11.7%), whereas the fetal birth-weight decreased significantly (2012: 3565 g vs. 2017: 3405 g, *p* < 0.05). In the group of diabetic pregnant women the percentage of cesarean sections, labour inductions and fetal birth defects was characterized by an insignificant upward trend. Risk of GDM was significantly increased in women aged over 35 years—(2012: OR 1.9 (95% CI: 1.1–2.9) and 2017: OR = 2.1 (95% CI: 1.5–2.9), *p* < 0.05—, as well as in overweight women—2012: OR 1.8 (95% CI: 1.2–2.7) and 2017: OR 2.6 (95% CI: 1.9–3.4), *p* < 0.05—during both analysed time periods. Based on the study results, it is necessary to develop population-based programmes to prevent obesity and to introduce and enforce the rules of appropriate screening for glucose tolerance disorders during pregnancy.

## 1. Introduction

### 1.1. Diabetes Mellitus—Overview

Diabetes mellitus (DM), commonly known as diabetes, is a group of metabolic disorders, whose common feature is a high blood glucose level over a prolonged period, being the result of absolute or relative insulin deficiency [1,2]. Detrimental effect of the chronic hyperglycemia on metabolic pathways of proteins, lipids and electrolytes is well-established in the literature [2,3].

According to the Global Report on Diabetes published by the World Health Organization (WHO), in 2014 nearly 422 million of adults suffered from DM worldwide [4]. In addition, recent research performed by the American Diabetes Association (ADA) revealed that 1.5 million Americans are diagnosed with DM every year [5].

### 1.2. Diabetes Mellitus in Pregnancy—Classification

DM in pregnancy may be divided into two major sub-types. One of them is pregestational DM (PGDM) which preexists in women who get pregnant [2,3,6]. The second one, hyperglycemia which was first detected during pregnancy should be categorized using the WHO criteria as DM in pregnancy (DIP) or gestational DM (GDM) [4,7]. According to the current standards, DIP is a condition that may be diagnosed, if standard DM criteria are met during screening, whereas GDM is diagnosed when women meet at least one of the criteria during 75g oral glucose tolerance test (OGTT) [3,7,8]. GDM occurs in pregnant women who develop hyperglycemia, but do not have a previous history of diabetes [2,3,9].

According to DeSisto et al. the prevalence of GDM in 2010 was reported at 4.6% when analysed using birth certificates and reached 8.7% when reported by standardized questionnaires [10]. Another study from the United States conducted by Fong et al. revealed the prevalence of GDM of 5.34%, while the PGDM equaled 0.82% [11].

It is of importance that women with DM and GDM are at an increased risk of multiple complications during pregnancy and at delivery [12,13,14].

### 1.3. Obesity and Obesity in Pregnancy—Overview

Obesity is a pathological condition characterized by an excessive accumulation of fat [15]. Body mass index (BMI) is the most popular tool used in obesity classification [16]. According to current standards, obesity is diagnosed when patient’s BMI exceeds 30 kg/m^2^ [16,17]. Most commonly obesity is caused by an excessive energy intake, lack of physical activity in conjunction with epigenetic and genetic predispositions [18,19,20]. According to Global Burden of Diseases 2015 Obesity Collaborators study including data from 195 countries, obesity occurs in about 600 million of adults and, what is even more worrying, in almost 100 million of children and adolescents [18]. During pregnancy obesity has a major impact on both maternal metabolism and fetal development [21]. The metabolic pathways that are most commonly altered in obese patients include glucose metabolism, increased insulin resistance and disrupted fat oxidation [21]. According to a recent study by Chen et al. in 2014 the estimated number of overweight and obese pregnant women equaled 38.9 million, with only obese women accounting for 14.6 million [22]. In the course of pregnancy obese women are at an increased risk of multiple perinatal complications, including preeclampsia, GDM, DIP and others [23,24,25,26].

### 1.4. Diabetes and Obesity during Pregnancy—Polish Guidelines and Standards

Education, optimal self-management and medical personnel support are crucial to prevent serious complications and reduce the risk of long-term complications [7,27]. Different countries developed various guidelines adapted to their systems of healthcare. Accordingly, Poland has its own guidelines concerning hyperglycemia in pregnancy. Interestingly, nowadays in Poland three documents tackle the issue of standardization and guidelines for hyperglycemia in pregnancy. They were developed by the Polish Society of Gynecologists and Obstetricians (PSGO) [2], Diabetes Poland [3] and by the Ministry of Health of Poland [28]. According to PSGO and Diabetes Poland the categorization and the diagnostic criteria of hyperglycemia during pregnancy are in accordance with WHO guidelines. Both documents advise screening in the first as well as in the late second or early third trimester of gestation [2,3]. The new standards of healthcare during pregnancy issued by the Polish Ministry of Health in 2018 include the recommendation to perform standard screening in the first trimester or at the time of the first obstetric visit. However, the second round of screening differs, as it should be performed between the 24th and 26th week of gestation, instead of the 24–28th week period as recommended by the WHO [28].

Pregnancy management practice in Poland changed significantly between the years 2012 and 2017. According to a 2012 standard by the Polish Ministry of Health on pregnancy management a single fasting glucose measurement should be performed in the first trimester, whereas in the late second/early third trimester it is advised to perform a two-step 75 g OGTT for the diagnosis of GDM [29]. Due to subsequent clinical trial results, some changes were introduced and many clinicians started to use new WHO guidelines (2013) [4,7]. The Hyperglycemia and Adverse Pregnancy Outcome (HAPO) study included a recommendation of a three-step 75 g OGTT as a new equalization criterion [30]. PSGO and Diabetes Poland guidelines were both up-to-date and evolved annually in case of Diabetes Poland [31], and in case of PSGO the guidelines appeared in 2011 [32], were updated in 2014 [33] and then re-edited and updated in 2017 [2]. As a result, in 2017, the current strategy for women in Poland included fasting glucose assessment in the first trimester and a three-step 75 g OGTT performed between 24th and 28th pregnancy weeks [2].

PSGO also published their own guidelines concerning perinatal care over obese pregnant women in year 2012 [34]. Importantly, as regards women with BMI ≥ 30 kg/m^2^, 75 g OGTT was recommended as early as the first trimester of pregnancy, instead of a single fasting glucose measurement performed in the general obstetric population [34].

Numerous recommendations published over the years show how difficult it is to manage pregnant women with DM or obesity. The aim of the present study was to compare the perinatal outcomes in the populations of diabetic and obese Polish women, and those with both conditions, based on the results of a national survey performed in years 2012 and 2017, as well as to determine the risk factors of GDM. We would like to evaluate how those outcomes and correlated measurements changed with the implementation of the new guidelines as standards.

## 2. Materials and Methods

The analyses of pregnant women were carried out in years 2012 and 2017 within the Polish Pregnancy-related Assessment Monitoring System (Pol-PrAMS). This population-based study was conducted in all of the hospitals in Poland. Groups of Polish women and their newborns were surveyed during postpartum hospitalization. Thus, all of the women hospitalized postpartum on the designated days of the study were deemed eligible for the study. Informed consent was verbally obtained from all women, which was approved by the Ethics Committee. Participation was anonymous and voluntary, and the surveys were completed by the women after consent. Thus, each completed questionnaire was a documentation of consent to the study.

The survey was carried out once in each hospital, which had at least one of the following units in its structure: maternity ward, department of gynecology and obstetrics, department of obstetrics with rooming-in, labor ward or neonatal department. The survey was conducted simultaneously throughout the country, using the structures of the Poviat Sanitary and Epidemiological Stations, as units subordinate to the Chief Sanitary Inspectorate. These types of Stations are located in every poviat in Poland (poviat is the second level of the administrative division of Poland), which allowed for the efficient conduction of research throughout the country within a few weeks of the year. In 2012 the study was conducted on one day in each hospital, during the third week of March. In 2017 the study was conducted between the 2 February and 22 March.

The evaluation was preceded by obtaining the consent from principals of each hospital. In 2012, 3555 mothers were hospitalized in 395 units on designated days for the study. The consent to conduct the study was obtained from the directors of 377 institutions where 2905 mothers were hospitalized. A total of 2825 questionnaires were qualified for the statistical analysis. In 2017, births took place in 397 hospitals and consent was obtained from 380 directors. A total of 3627 women were hospitalized and 3451 questionnaires were qualified for the statistical analysis.

The questionnaire was divided into two parts. The first part contained 77 questions concerning: maternal age, place of residence, education, marital, social and economic status, maternity profile (i.e., earlier births, miscarriages, possible difficulties in conceiving), as well as risky health behaviours prior to and during pregnancy (e.g., smoking, alcohol, drugs and other psychoactive substance abuse). This part also contained data on the course of pregnancy (i.e., reasons for hospitalization, pregnancy complications, diagnostic tests performed during pregnancy). There was no defined framework for information on socio-economic data. They were the subjective opinion of women themselves. However, social conditions can be described as not only features of individuals and households, such as income, wealth, educational attainment, family structure, housing, and transportation resources, but also features of communities, such as the prevalence and depth of poverty, rates of crime, accessibility of safe places to play and exercise, availability of transportation to jobs that provide a living wage, and availability of good schools and sources of nutritious food in a neighborhood [35]. Mothers who stayed in hospitals after birth completed the first part of the survey. The second part had nine questions that were filled-in by the medical personnel providing healthcare to the mother and newborn, with the use of medical records (pregnancy cards and patient’s medical history). The questions in this part concerned the mode of delivery, newborn’s health status after birth and birth defects. It also included questions about the results of laboratory tests performed on mothers and newborns after birth. The design of Pol-PrAMS study is presented in detail in another paper [36].

The survey aimed to compare the obstetric outcomes in a group of women with the diagnosis of DM and obesity, and to determine the risk factors of GDM. Women with BMI ≥ 25 kg/m^2^ or ≥30 kg/m^2^ were defined as overweight or obese, respectively. BMI was calculated on the basis of data, such as height and weight before pregnancy, provided by the women in the survey. Currently in Poland, the diagnosis of GDM is based on the three-step 75 g OGTT (fasting glucose ≥ 92—5.1 mmol/L; in 60 min ≥ 180 mg/dL—10 mmol/L and/or in 120 min ≥ 153 mg/dL—8.5 mmol/L]) [2]. These were also the criteria for the diagnosis of GDM in year 2017. In 2012, GDM was diagnosed based on the recommendations of the Polish Ministry of Health [29]. According to the standard on pregnancy management, GDM diagnosis was based on the two-step 75 g OGTT (fasting glucose ≥ 100 mg/dL—5.5 mmol/L—and/or in 120 min ≥ 140 mg/dL—7.8 mmol/L) performed between 24–28 gestational weeks.

### Statistical Analysis

Overall, 2825 women in year 2012 and 3451 women in year 2017 were included in the study. Continuous variables were compared using a Student’s *t*-test, while a chi-square test was applied for categorical variables. The results were expressed as the mean and standard deviation, or as a frequency (%). Logistic regression models were created to estimate the odds ratios (OR) and 95% confidence intervals (95% CI) for associations between selected variables and the risk of GDM development. All statistical analyses were performed using IBM SPSS software version 25 (IBM, Armonk, NY, USA). A *p*-value of < 0.05 was considered statistically significant.

The aim of the study was to compare the obstetric outcomes in groups of women with the diagnosis of DM and obesity, and to determine the risk factors of GDM. Therefore, the groups of women were standardized in terms of different ages and places of residence in both populations. As a result, we minimized the chance of bias due to differences in the structure of two analysed populations of women. Supplementary Table S1 presents the raw characteristics of the groups of women prior to the standardization. Further analyses were performed with the use of weighted data, in which the structure of the population studied in 2012 was matched to the population studied in 2017 in terms of the age structure and place of residence. The matching was carried out with the rim weighting method (SPSSINC RAKE procedure). The analysed proportions, before and after weighting, did not reveal statistically significant differences as regards the place of residence. The implemented method involves the calculation of the specific weight for each record, so a value larger than “one” may be attributed to a single record. It also provides explanation for the differences in the numbers of subjects in each group during both study periods. Table 1 presents the characteristics of women in both groups after the standardization.

## 3. Results

Following the standardization, the age structure was similar in both groups of participants with the highest percentage of women being aged over 30 years. Women who completed tertiary education constituted the highest percentage of participants in both analysed groups—48.8% in 2012 and 51.3% in 2017. The rates of women with the primary education were the lowest: 6.1% and 5.6% in years 2012 and 2017, respectively. The largest percentage of women in both groups lived in rural areas: 39.3% in 2012 and 41.2% in 2017. Slightly over 34% of participants in both groups lived in towns/cities with up to 100,000 inhabitants. The largest percentage of women in both groups described their social and economic conditions as good. Normal body weight expressed as BMI 18.5–24.99 kg/m^2^ was noted in 69.5% of women in 2012, while in 2017 the respective percentage reached 66.5%. Overweight women constituted 16.7% and 18.0%, while obese 5.5% and 7.5% of studied population in years 2012 and 2017, respectively (*p* < 0.05). The mean height of women in both groups was approx. 166 cm. A slightly increased gestational weight gain was observed in the group of women who gave birth in 2012. It reached 15.5 kg, while in 2017 it was 14.7 kg, (*p* < 0.05). Both groups of women differed significantly with respect to pregestational BMI, gestational weight gain, social conditions and the economic status (*p* < 0.05). Table 1 presents the characteristics of women in both analysed years.

The percentage of women whose pregnancy was complicated by DM increased significantly over the 5-year period (*p* < 0.05) (Table 2). The respective percentages for the years 2012 and 2017 amounted to 4.5% and 7.2% of studied population. GDM prevailed in both analysed years. It accounted for 89.0% of DM cases in 2012 and 85.6% in 2017. The percentage of women in whom GDM was diagnosed in the 1st trimester of pregnancy increased significantly from 3.5% in 2012 to 13.6% in 2017 (*p* < 0.05).

Both analysed populations differed in terms of the percentage of women who had been obese before the pregnancy. In 2012 women with BMI ≥ 30 kg/m^2^ constituted 5.5%, while in 2017—7.5% of the total study group (*p* < 0.05). The differences were also observed in rates of obese women in whom the diagnosis of GDM was made during the pregnancy. The respective percentages were 0.3% in 2012 and 0.9% in 2017 (*p* < 0.05). The distribution of diabetes and obesity in the study populations is presented in Table 2.

Table 3 presents selected perinatal outcomes in individual groups of women in the years 2012 and 2017. No statistically significant differences were noted in terms of diagnosed fetal defects. The comparison of variables in 2012 and 2017 revealed an insignificant increase in the percentage of congenital defects in the offspring of women suffering from DM (0.0% vs 1.0%). On the contrary, the percentage of birth defects decreased in obese pregnant women (3.3% vs. 2.1%). Between the years 2012 and 2017 a significant increase in the percentage of induced deliveries and instrumental deliveries/cesarean section (CS) in the whole study group was noted (*p* < 0.05). At the same time, no changes referring to the fetal birth-weight were observed in the total study population.

In the group of obese women the percentage of induced deliveries increased insignificantly over the analysed 5-year period (9.9% vs. 11.7%), whereas the fetal birth-weight decreased significantly (3565 g vs. 3405 g, *p* < 0.05). In 2012 almost 45% of obese women with concurrent GDM delivered via CS or with the use of a vacuum extractor or obstetric forceps. The respective percentage in 2017 was 64.5%. The percentage of induced deliveries in this subgroup of patients was comparable in both study periods (approx. 22%). The mean fetal birth-weight reached 3603 g and 3419 g, in years 2012 and 2017, respectively. However, the difference was not statistically significant.

In the group of women with a history of DM the percentage of all the perinatal outcomes was characterized by an upward trend over the years. However, statistically significant differences were not observed (Table 3). The exact levels of statistical significance were as follows: type of delivery, *p* = 0.56; labor induction, *p* = 0.32; fetal birth defects, *p* = 0.26 and fetal birth-weight, *p* = 0.59.

**Figure 1:**
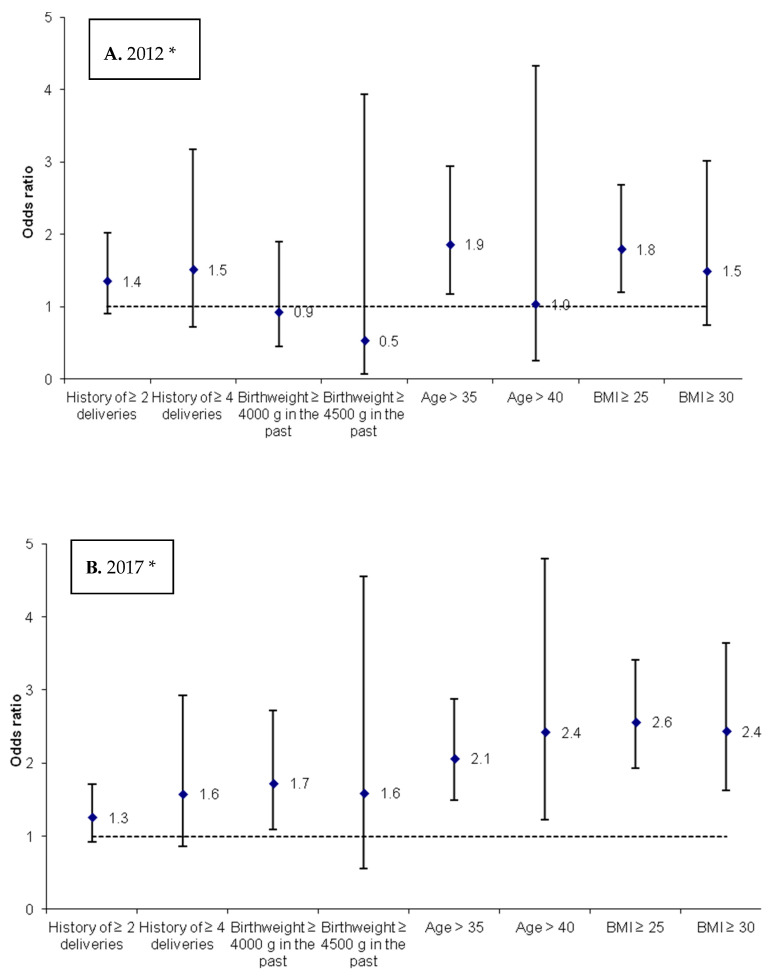
Risk factors of gestational diabetes mellitus in years 2012 (**A**) and 2017 (**B**). (* a group of women belonging to the complementary category of an individual variable was a reference category for each of the variables).

Figure 1 presents the risk of developing GDM in the years 2012 and 2017 depending on selected variables. In both study periods the risk of GDM occurrence was not increased in case of at least 2 or 4 deliveries in the past, as well as in the group of women who delivered fetuses with the birth-weight equal to or higher than 4500 g. Additionally, in 2012 the risk was not increased in a group of women who delivered fetuses weighing 4000 g or more, women aged over 40 years and with pregestational BMI exceeding 30 kg/m^2^. A statistically significant increase in the risk of GDM was observed in women aged over 35 years both in 2012 (OR = 1.9, 95% CI: 1.1–2.9) and in 2017 (OR = 2.1, 95% CI: 1.5–2.9). Moreover, an increased risk of GDM was noted in women with the pregestational BMI exceeding 25 kg/m^2^ in both study periods. In 2012 the OR was 1.8 (95% CI: 1.2–2.7), whereas in 2017 the OR was 2.6 (95% CI: 1.9–3.4). As regards the population studied in 2017 a significant increase in the risk of GDM was observed in those women who had delivered fetuses with the birth-weight of 4000 g or more (OR = 1.7, 95% CI: 1.1–2.7), aged over 40 years (OR = 2.4, 95% CI: 1.2–4.8) and with pregestational BMI ≥ 30 kg/m^2^ (OR = 2.4, 95% CI: 1.6–3.6).

## 4. Discussion

In the present study we investigated perinatal outcomes in a population of Polish women based on the results of a national survey conducted in the years 2012 and 2017. The analysis of data obtained over this 5-year period indicated a significant increase in the percentages of pregnancies complicated by obesity and DM. At the same time, national recommendations published in years 2011/2012 together with their later updates, concerning the management of obese and/or diabetic pregnant women contributed to the significant reduction in the fetal birthweight in the first group of patients and an increased detectability of GDM in the first trimester of gestation [2,32,33,34]. Both the patient’s age >35 at the moment of conception and pre-gestational BMI >25 kg/m^2^ constituted significant risk factors for developing GDM in both analysed time periods.

The observed upward trend referring to the percentage of CS, patients with pre-pregnancy BMI exceeding 30 kg/m^2^, and pregnancies complicated by GDM/PGDM in the population of Polish women reflects worldwide tendencies [22,37,38,39,40]. The common risk factor for the above-mentioned complications is the growing maternal age at conception associated with a 2- to 3-fold higher perinatal morbidity [41]. Apart from the higher rate of CS and GDM, a positive correlation between the advanced maternal age and the increased risk of fetal chromosomal aberrations, preterm delivery, stillbirth, multiple pregnancy, pre-eclampsia, thrombosis, postpartum hemorrhage, hysterectomy and stroke was noted [41,42]. In the Polish population in year 2017, over 46% of women were over 30 years of age, while 5 years before the respective percentage was slightly over 32% (see: Supplementary Table S1). The observed changes in the age structure of Polish pregnant women indicate the necessity of updating domestic recommendations concerning perinatal care with particular attention paid to suitable prophylaxis, diagnostics and treatment in patients older than 35 years. To date in Poland only two documents have been published aiming to improve perinatal care in case of advanced maternal age: PSGO recommendations concerning the indications for induced delivery in patients over 40 years issued in 2017 [43] and the programme of prenatal tests covered by the National Health Fund for patients older than 35 years which has been in operation since 2005 [44].

Based on the results of the national survey in 2017 in Poland the percentage of CS amounted to 42% and exceeded the “ideal rate” suggested by the WHO by almost 30% [38,45]. Apart from the ageing of the obstetric population and its related morbidity, the main reasons for the increasing number of CS in Poland include wide list of indications for surgical deliveries, as well as the preferences of women themselves. Direct and indirect interrelations between those factors create an urgent necessity for developing new or updating the already-existing national strategies concerning the indications for CS. PSGO recommendations issued in 2018 constitute the response to the above-mentioned demands and include the majority of obstetric and extra-obstetric indications for a surgical intervention during pregnancy [46]. Nonetheless, the assessment of their effectiveness requires a population-based survey to be conducted in subsequent years.

Over the 5-year period the percentage of overweight and obese women in Poland increased from 16.7% to 18% and from 5.5% to 7.5%, respectively. According to the literature, an increased caloric supply, urbanization and gross national income are the main factors responsible for the excessive weight gain and obesity in pregnant women in high-income countries [22]. Polish data grossly confirmed the worldwide observations. The comparison of national survey performed in 2017 with data obtained in 2012 showed a significant increase in the percentage of women with tertiary education and those who described their social conditions and economic status as very good (see: Supplementary Table S1). At the same time no differences were observed with regard to the place of residence with the percentage of women living in cities with over 100,000 inhabitants at around 25% in both analysed time periods.

Observed tendencies revealed through the national survey are unfavorable, as they indicate a strong negative trend towards the increased rate of obese women over the past few years despite a significant improvement concerning the level of education and the socioeconomic status (see: Supplementary Table S1). Although large population-based studies concerning the socioeconomic risk factors of obesity have not been conducted in the Polish population so far, research performed by other authors demonstrated their strong inter-relations. In the study by Cutler et al. authors observed the positive correlation between years of schooling and a reduced risk of being overweight and obese. Simultaneously, persons with higher levels of education were more physically active. The relationship between the level of education and obesity seems to be non-linear with the increasing effects of additional years of schooling [47]. In addition, a negative association between the level of education and the probability of being overweight was demonstrated in a cross-sectional study of twins [48]. According to some authors’ suggestions, the patient’s sex significantly modifies the association between socioeconomic factors and obesity. Among women, a higher level of education presented the strongest correlation with low BMI and waist-to-hip ratio (WHR), whereas, in men both parameters were more affected by the income [49].

Despite the unfavorable upward tendency referring to the percentage of pregnancies with concomitant obesity, the recommendations published by PSGO in 2012 contributed to the improvement in perinatal care in the affected group of patients, i.e., obese women [34]. The effectiveness of the measures taken is reflected by the significant decrease in the neonatal birthweight and the simultaneous increase in the number of induced deliveries. The observed changes regarding perinatal outcomes are directly associated with the recommendations of a Polish group of experts in which they emphasized the necessity and determined the aims of the preconception care of obese patients, set the upper limit of weight gain during pregnancy as well as suggested performing an additional pre-delivery ultrasound examination to estimate the fetal birth-weight. Furthermore, despite the fact that isolated obesity does not constitute direct indication for labor induction, such management was suggested after 38 gestational weeks in patients with BMI over 40 kg/m^2^. As regards the main assumptions of the perinatal care in obese pregnant women Polish recommendations are similar to those published by other national obstetric societies [50]. Nonetheless, issues such as folic acid and vitamin D supplementation, the prophylaxis of venous thromboembolism or pre-eclampsia require updating.

One of the assumptions of the recommendations published by PSGO was the proper control of body weight in pregnancy [2,32,33]. Polish recommendations remained in line with international guidelines, which indicate that a woman with a normal body weight expressed in BMI should gain between 11.5 kg and 15.9 kg during pregnancy [51]. As a consequence, it was possible to reduce the average weight gain in pregnancy from 15.5 kg in 2012 to 14.7 kg in 2017. Noteworthy, in year 2012 the average weight gain of pregnant women in Poland was close to the specified upper limit.

Certain concerns are raised as regards the group of obese pregnant women in whom GDM was diagnosed. This population of patients noted significant increase from 0.3% to 0.9% over the 5-year period. Moreover, despite more effective perinatal care leading to reduced neonatal birthweight, the percentage of induced deliveries did not change, whereas the percentage of CS increased by almost 20% in the group of women with BMI > 30 kg/m^2^ and concomitant GDM. The association between obesity/GDM and an increased risk of an operative delivery is well-documented and, most probably, the observed tendencies in the Polish population are related to the synergistic effect of both diseases [52,53,54]. Nevertheless, due to the increasing percentage of CS performed in Poland, publication of separate recommendations for labor induction in obese diabetic women seems to be justified.

Apart from the advanced maternal age, obesity is one of the most important risk factors of GDM, which was confirmed by the results of the Polish national survey. Data obtained in the years 2012 and 2017 reflect the worldwide tendency as regards the increased incidence of GDM [55,56]. From the clinical perspective the 4-fold increase in GDM detectability in the first trimester constitutes one of the most important observations. The presumable reason for that is the increasing awareness of healthcare providers regarding diagnostic standards of DM in pregnancy. According to Polish recommendations, patients presenting the risk factors of GDM (including obesity) should be offered a three-step 75 g OGTT during the first antenatal visit. Thus, the increasing frequency of OGTTs performed in the 1st trimester of gestation may be associated with an increased detectability of hyperglycemia in early pregnancy observed in year 2017 as compared to 2012. An increased GDM detectability in the 1st trimester of pregnancy seems to be a favorable phenomenon, as it provides early care to patients with glucose tolerance disorders, which were undetected prior to gestation. A large multicenter DALI trial revealed that patients with an early GDM diagnosis are characterized by a significantly poorer metabolic profile as compared to those diagnosed in the second/third trimester. An early intervention may therefore contribute to the better glycemia control, reduced gestational weight gain and ultimately reduced neonatal adiposity [57].

The main limitation of our study is the fact that diagnostic criteria for gestational diabetes mellitus have changed between 2012 and 2017. Nevertheless, it allowed us to assess the impact of the new diagnostic strategy on the perinatal outcomes. Another limitation is the occurrence of differences in the characteristics of analysed cohorts of women in both study periods. We tried to eliminate this bias using rim weighting method (SPSSINC RAKE procedure). Limitation is also the retrospective character of our study in conjunction with the voluntary participation. In addition, some of the variables evaluated in the study relied on the subjective assessment of study participants, such as the assessment of socio-economic conditions.

Despite that, the present study is the most extensive analysis of the perinatal outcomes in obese and diabetic pregnant women in Poland conducted so far. It’s undeniable advantage is associated with the size of the study group and the fact that it was performed in the majority of Polish obstetric centers. As a consequence, it minimized the risk of selection bias, which might be due to the fact that the participation in the study was voluntary. Furthermore, the clinical value of the study is increased by the fact that the data collection was performed over analogous time periods with a 5-year interval. The latter facilitated the analysis of the trends in the epidemiology of obesity and diabetes in the population of Polish pregnant women. Study results reflected tendencies observed in many developed countries as regards the increasing frequency of obesity and GDM, as well as the fact that women of reproductive age decide to conceive later. It seems, however, that apart from the increased rate of CS, the above-mentioned trends did not have significant impact on the other adverse perinatal outcomes. Nonetheless, it is necessary to develop population-based programmes to prevent obesity and to introduce and enforce the rules of appropriate screening for glucose tolerance disorders during pregnancy.

## 5. Conclusions

Over the 5 years period a significant increase in the percentage of obese women of reproductive age was observed in Poland. This fact, along with the change in the diagnostic criteria for GDM, contributed to the increase in the percentage of diabetes diagnoses, in particular in the first trimester of pregnancy. Noteworthy, in the population of obese women we observed a significant decrease in the fetal birthweight. In the analysed period of time, several documents appeared in Poland that defined the standards of care for pregnancies complicated by diabetes and obesity. These recommendations provide the most probable explanation of the observed national trends.

## Figures and Tables

**Table 1:** Characteristics of the study population.

Characteristics	2012			2017		*p*
	*N*	*%*		*N*	%	
**Age** (years)						ns
≤25	543	19.2		656	19.2	
26–30	974	34.5		1177	34.5	
Over 30	1307	46.3		1580	46.3	
**Education**						ns
Primary	167	6.1		187	5.6	
Secondary	1240	45.1		1431	43.1	
Tertiary	1341	48.8		1704	51.3	
**Place of residence** (inhabitants)						ns
City (≥100,000)	725	25.8		804	24.1	
Town/city (<100,000)	977	34.8		1154	34.7	
Rural area	1103	39.3		1373	41.2	
**Social conditions**						<0.05
Very good	735	26.0		1295	37.8	
Good	1610	57.0		1821	53.2	
Average/poor	478	16.9		308	9.0	
**Economic status**						<0.05
Very good	284	10.1		652	18.9	
Good	1675	59.4		2188	63.4	
Average/poor	863	30.6		611	17.7	
**BMI before pregnancy** (kg/m^2^)						<0.05
<18.5	228	8.3		267	8.0	
18.5–24.99	1907	69.5		2211	66.5	
25.0–29.99	458	16.7		599	18.0	
≥30	151	5.5		247	7.5	
**Parameter**	***N***	**Mean**	**S.D.**	**Minimum**	**Maximum**	***p***
**Height** (cm)						ns
2012	2752	166.0	6.1	139.0	188.0	
2017	3344	165.9	5.9	146.0	198.0	
**Gestational weight gain** (kg)						<0.05
2012	2753	15.5	5.7	0.0	102.0	
2017	3307	14.7	6.2	0.0	89.0	

BMI—body mass index; ns—non-significant, *p* > 0.05.

**Table 2:** The distribution of diabetes mellitus and obesity in Polish pregnant women in years 2012 and 2017 (the numbers in brackets refer to the percentage of GDM/PGDM subtypes in all patients diagnosed with diabetes mellitus).

Variable	2012		2017		*p*
	*N*	%	*N*	%	
**Diabetes** (total number of patients)	2799	100	3451	100	
**No**	2672	95.5	3201	92.8	<0.05
**Yes**	127	4.5 (100.0)	250	7.2 (100.0)	
PGDM	14	0.5 (11.0)	36	1.0 (14.4)	
GDM	113	4.0 (89.0)	214	6.2 (85.6)	
**Period when GDM was diagnosed**					<0.05
1st trimester	4	3.5	29	13.6	
2nd or 3rd trimester	109	96.5	185	86.4	
**Obese** (BMI ≥ 30 kg/m^2^)					<0.05
**No**	2592	94.5	3077	92.6	
**Yes**	151	5.5	247	7.5	
**Obese with GDM**					<0.05
**No**	2795	99.7	3409	99.1	
**Yes**	9	0.3	32	0.9	

PGDM—pregestational diabetes mellitus; GDM—gestational diabetes mellitus; BMI—body mass index.

**Table 3:** Selected perinatal outcomes in the individual groups of women analysed in years 2012 and 2017.

Variable	All Women			Obese Women *			Obese * with GDM			Diabetes **		
	2012 *N* (%)	2017 *N* (%)	*p*	2012 *N* (%)	2017 *N* (%)	*p*	2012 *N* (%)	2017 *N* (%)	*p*	2012 *N* (%)	2017 *N* (%)	*p*
**Type of delivery**			<0.05			ns			ns			ns
Vaginal	1706 (61.5)	1879 (58.0)		71 (48.0)	109 (48.0)		5 (55.6)	11 (35.5)		72 (58.5)	134 (55.4)	
C-section/assisted	1070 (38.5)	1360 (42.0)		77 (52.0)	118 (52.0)		4 (44.4)	20 (64.5)		51 (41.5)	108 (44.6)	
**Labor induction**			<0.05			ns			ns			ns
No	2563 (90.8)	3069 (88.9)		136 (90.1)	218 (88.3)		7 (77.8)	25 (78.1)		112 (88.2)	211 (84.4)	
Yes	261 (9.2)	382 (11.1)		15 (9.9)	29 (11.7)		2 (22.2)	7 (21.9)		15 (11.8)	39 (15.6)	
**Fetal birth defects**			ns			ns			-			ns
No	2785 (98.6)	2735 (98.5)		146 (96.7)	185 (98.9)		9 (100.0)	24 (100.0)		127 (100.0)	202 (99.0)	
Yes	39 (1.4)	42 (1.5)		5 (3.3)	2 (1.1)		0 (0.0)	0 (0.0)		0 (0.0)	2 (1.0)	
**Fetal birth-weight** (g)	3373	3368	ns	3565	3405	<0.05	3603	3419	ns	3285	3342	ns
Standard deviation	572	567		679	575		516	608		640	615	
Minimum	380	300		1200	695		2900	1720		870	338	
Maximum	5220	5470		5220	4870		4550	4850		4550	5000	

ns—non-significant, *p* > 0.05; * body mass index before pregnancy ≥30 kg/m^2;^ ** diagnosis of pregestational or gestational diabetes mellitus in the current pregnancy.

## Data Availability

The data presented in this study are available on request from the corresponding author. The data are not publicly available due to privacy.

## Supplementary Materials

The following are available online at https://www.mdpi.com/1660-4601/18/2/560/s1, Table S1: Characteristics of the study population—raw data.
