# Supplementary material for: Perinatal Outcomes in a Population of Diabetic and Obese Pregnant Women—The Results of the Polish National Survey
Source: Int J Environ Res Public Health. 2021 Jan 11;18(2):560. doi: 10.3390/ijerph18020560 (PMC7827210; doi:10.3390/ijerph18020560)
Supplement: Supplementary file 1 [file ijerph-18-00560-s001.pdf]

**Table S1.** Characteristics of the study population—raw data.

| Characteristics | 2012 | | 2017 | | p |
|---|---|---|---|---|---|
| | N | % | N | % | |
| **Age** (years) | | | | | <0.05 |
| ≤25 | 833 | 29.5 | 656 | 19.2 | |
| 26– 30 | 1085 | 38.4 | 1177 | 34.5 | |
| Over 30 | 906 | 32.1 | 1580 | 46.3 | |
| **Education** | | | | | <0.05 |
| Primary | 191 | 6.8 | 187 | 5.5 | |
| Secondary | 1284 | 45.5 | 1431 | 42.3 | |
| Tertiary | 1283 | 45.4 | 1704 | 50.3 | |
| Other | 67 | 2.3 | 64 | 1.9 | |
| **Place of residence (inhabitants)** | | | | | ns |
| City (≥100,000) | 703 | 25.0 | 804 | 24.1 | |
| Town/city (<100,000) | 945 | 33.7 | 1154 | 34.7 | |
| Rural area | 1159 | 41.3 | 1373 | 41.2 | |
| **Social conditions** | | | | | <0.05 |
| Very good | 731 | 25.9 | 1295 | 37.8 | |
| Good | 1600 | 56.6 | 1821 | 53.2 | |
| Average/poor | 494 | 17.5 | 308 | 9.0 | |
| **Economic status** | | | | | <0.05 |
| Very good | 284 | 10.1 | 652 | 18.9 | |
| Good | 1637 | 57.9 | 2188 | 63.4 | |
| Average/poor | 904 | 32.0 | 611 | 17.7 | |
| **BMI before pregnancy** (kg/m$^2$) | | | | | <0.05 |
| <18.5 | 252 | 9.2 | 267 | 8.0 | |
| 18.5–24.99 | 1915 | 69.7 | 2211 | 66.5 | |
| 25.0–29.99 | 444 | 16.2 | 599 | 18.0 | |
| ≥30 | 137 | 4.9 | 247 | 7.5 | |

ns—non-significant, $p > 0.05$.
